# Pulmonary mycobacteriosis caused by *Mycobacterium peregrinum*: A case report

**DOI:** 10.1097/MD.0000000000028809

**Published:** 2022-02-11

**Authors:** Yutaka Morita, Yoshihiro Kondo, Eiji Takeuchi

**Affiliations:** a Division of Pulmonary Medicine, National Hospital Organization Kochi Hospital, 1-2-25 Asakuranishimachi, Kochi city, Kochi, Japan; b Department of Internal Medicine, Japanese Red Cross Kochi Hospital, 1-4-63-11 Hadaminamimachi, Kochi city, Kochi, Japan; c Department of Clinical Investigation, National Hospital Organization Kochi Hospital, 1-2-25 Asakuranishimachi, Kochi city, Kochi, Japan.

**Keywords:** *Mycobacterium peregrinum*, nontuberculous mycobacteria, pulmonary mycobacteriosis, rapidly growing nontuberculous mycobacteria

## Abstract

**Rationale:**

*Mycobacterium peregrinum* is a member of the group of rapidly growing nontuberculous mycobacteria. It mainly causes surgical site and catheter-related infections, while pulmonary infection is rare. We herein present a case of pulmonary infection caused by *M peregrinum*.

**Patient concerns:**

A 62-year-old woman visited our hospital with dyspnea and was admitted for the treatment of pneumonia in July 2018.

**Diagnosis:**

Chest computed tomography showed patchy opacities and consolidation in the bilateral lungs and a cavity in the right upper lobe, which persisted after the treatment of bacterial pneumonia 5 years ago. She was administered ceftriaxone and azithromycin. Consolidation in the bilateral lungs improved, whereas the cavity in the right upper lobe remained and the consolidation surrounding it gradually spread. On admission, the sputum acid-fast bacillus culture was positive, and *M peregrinum* was identified twice by mass spectrometry. The cavity and consolidation surrounding it were diagnosed as pulmonary mycobacteriosis caused by *M peregrinum*.

**Interventions:**

Although we recommended treatment for mycobacteriosis, the patient refused it.

**Outcomes:**

The patient is regularly followed up; however, the cavity wall is thickening and shadows have become mildly enhanced over the course of 3 years.

**Lessons:**

We herein present a rare case of pulmonary mycobacteriosis caused by *M peregrinum* and discuss the literature. Since limited information is currently available on pulmonary mycobacteriosis caused by *M peregrinum*, the accumulation of further case reports and the clarification of its clinical features are needed.

## 1 Introduction

Among nontuberculous mycobacteria (NTM), rapidly growing nontuberculous mycobacteria (RGM) are mainly reported as pathogens in lung, skin, soft tissue, and bone infections. They are generally classified into a number of groups including the *Mycobacterium fortuitum* group, *Mycobacterium chelonae abscessus* group, and *Mycobacterium mucogenicum* group. *Mycobacterium peregrinum* has been classified into the *M fortuitum* group and accounts for approximately 2% of RGM infections.^[1]^ Although it mainly causes surgical site and catheter-related infections, only a small number of cases have been reported to date. Lung infections caused by *M peregrinum* are even rarer and, thus, its clinical features remain unknown and there is currently no established treatment. We herein present a rare case of pulmonary mycobacteriosis caused by *M peregrinum*.

## 2 Case report

**Figure 1:**
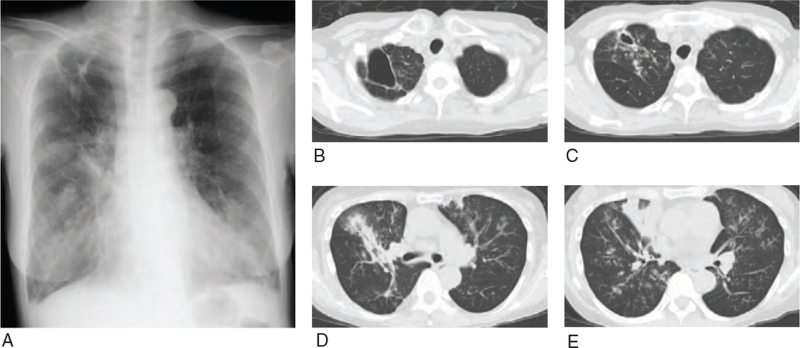
Chest X-ray (A) and chest computed tomography (CT) findings (B–E) on admission. A: Chest X-ray shows a cavity in the right upper lung field and consolidation in the bilateral lower lung fields. B–E: CT scanning shows a cavity in the upper right lobe, consolidation in the middle lobe, and patchy opacities in both lower lobes.

A 62-year-old woman visited our hospital with dyspnea in July 2018. She had a medical history of bronchial asthma and had inhaled budesonide/formoterol. In 2013, she had a negative sputum mycobacteria test and was treated for bacterial pneumonia. Her smoking history was 15 pack-years, and she was a current smoker. Her physical examination showed a body temperature of 36.8°C, heart rate of 119 beats per minute, blood pressure of 162/100 mm Hg, percutaneous oxygen saturation of 94% on 2 L/min oxygen with a nasal cannula, and respiratory rate of 28 breaths per minute. Chest auscultation revealed coarse crackles on both of the lower sides and normal heart sounds without murmurs. Laboratory examinations (Table 1) showed a white blood cell count of 11,860/μL with 89.1% neutrophils, hemoglobin of 13.3 g/dL, a platelet count of 30.4 × 10^4^/μL, blood urea nitrogen of 23 mg/dL, creatinine of 0.84 mg/dL, and C-reactive protein of 35.01 mg/dL. Chest X-ray revealed consolidation in the bilateral lower lung fields and a cavity in the right upper lung field (Fig. 1A). Chest computed tomography (CT) showed consolidation in the middle lobe, patchy opacities in both lower lobes, and a cavity with a diameter of 30 to 47 mm in the upper right lobe (Fig. 1B–E). There was no calcification or lymphadenopathy. The cavity in the right upper lobe remained after treatment for bacterial pneumonia in 2013. The patient was diagnosed with pneumonia and admitted to our hospital for treatment.

**Table 1:** Laboratory data on admission.

Hematology			Biochemistry		
WBC	11,860	/μL	AST	23	U/L
Neutro	89.1	%	ALT	26	U/L
Lymph	4.6	%	LDH	231	U/L
Mono	5.5	%	BUN	23	mg/dL
Eosino	0.0	%	Cre	0.84	mg/dL
Baso	0.8	%	Na	142	mEq/L
RBC	427 × 10^4^	/μL	K	4.2	mEq/L
Hb	13.3	g/dL	Cl	107	mEq/L
Plt	30.4 × 10^4^	/μL	TP	6.4	mg/dL
			Alb	2.6	mg/dL
Serology					
CRP	35.01	mg/dL	Infection		
B-D-glucan	5.4	pg/mL	MAC Ab	0.72	U/mL

BUN = blood urea nitrogen, CRP = C-reactive protein.

**Figure 2:**
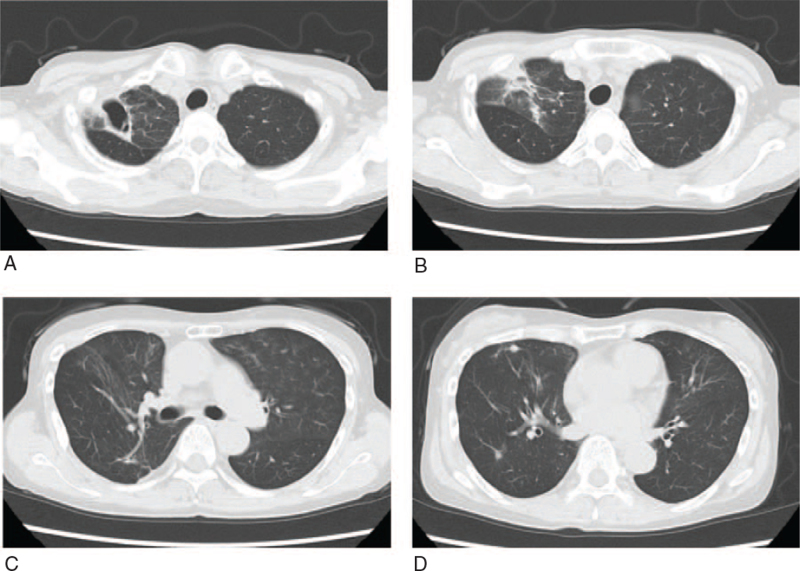
Chest computed tomography findings 3 months after diagnosis. A, B: Consolidation surrounding the cavity in the right upper lobe spread. C, D: Consolidation and patchy opacities in the bilateral lower lobes improved.

After a sputum culture test, antibiotic therapy was initiated with intravenous ceftriaxone (2 g per day) and oral azithromycin (500 mg per day). A bacterial sputum examination detected *Haemophilus influenzae* and *Klebsiella pneumoniae*. One of the 2 sputum mycobacterial smear tests was positive (Gaffky scale 2). Both sputum samples were subsequently positive in liquid cultures. *M peregrinum* was identified by mass spectrometry. Isolated *M peregrinum* showed a low minimal inhibitory concentration for levofloxacin and clarithromycin. Consolidation in both lungs improved with the administration of ceftriaxone for 15 days and azithromycin for 3 days; however, the cavity in the right upper lobe persisted. We decided to discharge the patient with follow-ups at the outpatient department. Three months after discharge, CT confirmed that consolidation surrounding the cavity in the right upper lobe had spread (Fig. 2). The patient was diagnosed with pulmonary mycobacteriosis caused by *M peregrinum*. Although the patient received a full explanation and we recommended treatment for mycobacteriosis, she refused this course of action. Therefore, we continue to periodically follow-up the patient. The cavity wall is thickening and the shadows have become mildly enhanced over the course of 3 years.

## 3 Discussion

RGM, called Runyon classification Group IV, are defined as species that colonize and achieve mature growth on pure cultures within 7 days. They are generally classified into 6 groups: the *M fortuitum* group, *M chelonae abscessus* group, *M mucogenicum* group, *Mycobacterium mageritense* group, *Mycobacterium smegmatis* group, and early pigmented rapidly growing mycobacteria. The *M fortuitum* group consists of *M fortuitum*, *M peregrinum*, *Mycobacterium houstonense*, and *Mycobacterium porcinum*. Since some cases of infection by the *M fortuitum* group have been reported together as infection by *M fortuitum*, only a few cases of *M peregrinum* have been described to date and, thus, its clinical features remain unclear.

The *M fortuitum* group is found in soil and water, including tap water and hospital water systems.^[2]^ To the best of our knowledge, there have been 20 case reports related to *M peregrinum* causing infections in surgical sites, cardiac devices, soft tissue, central catheters, and the lungs, including the present case (Table 2). Among these cases, 11 (55%) were male and the age at diagnosis ranged between 1 to 83 years. Elderly patients with pre-existing lung diseases, such as a previous history of lung tuberculosis and bronchiectasis, are susceptible to pulmonary mycobacteriosis by the *M fortuitum* group.^[22]^ However, a young man without any underlying diseases was also reported to have pulmonary infection caused by *M peregrinum*.^[18]^ There are no marked differences in symptoms from other NTM groups, such as chronic cough, sputum, fever, and weight loss. The CT findings of pulmonary mycobacteriosis by the *M fortuitum* group show multiple small nodules, patchy shadows, and bronchiectasis.^[23]^ Although cavity lesions are rare, 1 case in which infection caused by *M peregrinum* formed a cavity has been reported.^[20]^ In the present case, radiographic imaging revealed a cavity in the right upper lobe; however, it was not unclear whether this was due to *M peregrinum* infection or previous pneumonia. The *M fortuitum* group is particularly apt at contaminating water systems, and a previous study described mass infections of soft tissue from a foot bath.^[24]^ Therefore, it is diagnostically necessary to establish whether *M peregrinum* detected in just 1 sample is a contaminant or a pathogen. The American Thoracic Society/Infectious Diseases Society of America guidelines for NTM infections states that positive cultures need to be obtained from 2 separate samples of expectorated sputum.^[25]^ We diagnosed the present case from 2 positive sputum cultures and typical CT images. There have been 5 case reports of pulmonary mycobacteriosis caused by *M peregrinum*, and only 2 were diagnosed from 2 positive sputum samples.

**Table 2:** Previously reported cases of *M peregrinum* infection.

Case	Age/sex	Site of infection	Antibiotics	Ref
1	74/M	AICD	CPFX, CAM	^[3]^
2	59/F	Pacemaker	MFLX, S/T	^[4]^
3	75/M	Pacemaker	CPFX, CAM	^[5]^
4	17/F	Prosthetic aortic valve	CAM, AMK, IMP/CS, DOXY, S/T, RFP	^[6]^
5	38/M	Hickman catheter	VCM	^[7]^
6	2/M	Lymph node	CPFX, CAM, AMK	^[8]^
7	1/M	Lymph node	CPRM, IMP/CS	^[9]^
8	13/F	Skin and soft tissue	NA	^[10]^
9	58/F	Surgical site	LVFX, AMK, IMP/CS	^[11]^
10	30/F	Tonsillar abscess	CAM, IMP/CS, FRPM	^[12]^
11	83/M	Skin and soft tissue	MINO	^[13]^
12	45/M	Skin and soft tissue	SPFX, MINO	^[14]^
13	40/M	Skin and soft tissue	CPFX, CAM, AMK	^[15]^
14	30/F	Skin and soft tissue	CPFX, CAM, IMP/CS	^[16]^
15	40/F	Pneumonia	CPFX, CAM	^[17]^
16	24/M	Pneumonia	LVFX, CAM, EB	^[18]^
17	61/F	Pneumonia	LVFX, CAM, MINO	^[19]^
18	72/M	Pneumonia	EB, RFP, PZA, INH	^[20]^
19	68/M	Pneumonia	EB, RFP, PZA, INH	^[21]^
This case	62/F	Pneumonia	No treatment	

AICD = automatic implantable cardioverter defibrillator, AMK = amikacin, CAM = clarithromycin, CPFX = ciprofloxacin, CPRM = capreomycin, DOXY = doxycycline, EB = ethambutol, FRPM = faropenem, IMP/CS = imipenem/cilastatin sodium, INH = isoniazid, LVFX = levofloxacin, MFLX = moxifloxacin, MINO = minocycline, NA = not available, PZA = pyrazinamide, RFP = rifampicin, S/T = sulfamethoxazole/trimethoprim, SPFX = sparfloxacin, VCM = vancomycin.

The treatment of *M peregrinum* is not described in the official guidelines. Isolates of *M fortuitum* are generally susceptible to macrolides (clarithromycin and azithromycin), quinolones (ciprofloxacin, levofloxacin, and moxifloxacin), sulfonamides, linezolid, imipenem, doxycycline, cefoxitin, and aminoglycosides. In the treatment of lung disease caused by *M fortuitum*, at least 2 antibacterial agents from these agents with in vitro activity need to be administered for at least 12 months until sputum cultures become negative. *M fortuitum* isolates contain an inducible erm (39) gene that confers resistance to macrolides.^[26]^ Therefore, macrolides need to be administered with caution. Among 20 cases of *M peregrinum* infection, quinolones were used to treat 11 (55%), macrolides for 9 (45%), aminoglycosides for 6 (30%), carbapenems for 6 (30%), and tetracyclines for 4 (20%). In 5 cases of lung disease caused by *M peregrinum*, 2 were treated with rifampicin, isoniazid, pyrazinamide, and ethambutol,^[20,21]^ 1 with clarithromycin and ciprofloxacin,^[17]^ and 2 with clarithromycin, levofloxacin, and ethambutol/minocycline.^[18,19]^ Good treatment responses were achieved, excluding a case of polymyositis treated with infliximab. Since consolidation around the cavity gradually spread in the present case, we recommended treatment with 2 or more antibacterial agents, including the newer macrolides and quinolones. However, the patient refused this treatment. Therefore, we continue to periodically follow-up the patient. The cavity wall is thickening and the shadows have become mildly enhanced over the course of 3 years.

## 4 Conclusion

Limited information is currently available on pulmonary mycobacteriosis caused by *M peregrinum* and, thus, the accumulation of further case reports and the clarification of its clinical features are needed.

## Author contributions

**Conceptualization:** Eiji Takeuchi.

**Data curation:** Yutaka Morita, Yoshihiro Kondo.

**Formal analysis:** Yutaka Morita.

**Resources:** Yutaka Morita, Yoshihiro Kondo.

**Visualization:** Yutaka Morita.

**Writing – original draft:** Yutaka Morita.

**Writing – review & editing:** Eiji Takeuchi.

## References

[1] Brown-Elliott BA Wallace RJ Jr. Clinical and taxonomic status of pathogenic nonpigmented or late-pigmenting rapidly growing mycobacteria. Clin Microbiol Rev 2002;15:716–46. 12364376 10.1128/CMR.15.4.716-746.2002 PMC126856

[2] Galassi L Donato R Tortoli E. Nontuberculous mycobacteria in hospital water systems: application of HPLC for identification of environmental mycobacteria. J Water Health 2003;1:133–9. 15384724

[3] Short WR Emery C Bhandary M. Misidentification of *Mycobacterium peregrinum*, the causal organism of a case of bacteremia and automatic implantable cardioverter defibrillator-associated infection, due to its unusual acid-fast staining characteristics. J Clin Microbiol 2005;43:2015–7. 15815048 10.1128/JCM.43.4.2015-2017.2005 PMC1081373

[4] Chrissoheris MP Kadakia H Marieb M. Pacemaker pocket infection due to *Mycobacterium goodii*: case report and review of the literature. Conn Med 2008;72:75–7. 18306833

[5] Amraoui S Texier-Maugein J Bordachar P. PET scan in suspected but unproven pacemaker endocarditis. Arch Cardiovasc Dis 2012;105:125–6. 22424331 10.1016/j.acvd.2011.04.011

[6] Torres-Duque CA Díaz C Vargas L. Disseminated mycobacteriosis affecting a prosthetic aortic valve: first case of *Mycobacterium peregrinum* type III reported in Colombia. Biomedica 2010;30:332–7. 21713334

[7] Rodríguez-Gancedo MB Rodríguez-González T Yagüe G. *Mycobacterium peregrinum* bacteremia in an immunocompromised patient with a Hickman catheter. Eur J Clin Microbiol Infect Dis 2001;20:589–90. 11681443 10.1007/s100960100561

[8] Tsolia MN Chapgier A Taprantzi P. Disseminated nontuberculous mycobacterial infection in a child with interferon-gamma receptor 1 deficiency. Eur J Pediatr 2006;165:458–61. 16602008 10.1007/s00431-006-0110-7

[9] Koscielniak E de Boer T Dupuis S. Disseminated *Mycobacterium peregrinum* infection in a child with complete interferon-gamma receptor-1 deficiency. Pediatr Infect Dis J 2003;22:378–80. 12712974

[10] Pérez-Alfonzo R Poleo Brito LE Vergara MS. Odontogenic cutaneous sinus tracts due to infection with nontuberculous mycobacteria: a report of three cases. BMC Infect Dis 2020;20:295. 32316920 10.1186/s12879-020-05015-5 PMC7171849

[11] Nagao M Sonobe M Bando T. Surgical site infection due to *Mycobacterium peregrinum*: a case report and literature review. Int J Infect Dis 2009;13:209–11. 18848484 10.1016/j.ijid.2008.06.018

[12] Sakai T Kobayashi C Shinohara M. *Mycobacterium peregrinum* infection in a patient with AIDS. Intern Med 2005;44:266–9. 15805720 10.2169/internalmedicine.44.266

[13] Kamijo F Uhara H Kubo H. A case of mycobacterial skin disease caused by *Mycobacterium peregrinum*, and a review of cutaneous infection. Case Rep Dermatol 2012;4:76–9. 22548041 10.1159/000337825 PMC3339662

[14] Ishii N Sugita Y Sato I. A case of mycobacterial skin disease caused by *Mycobacterium peregrinum* and *M. scrofulaceum*. Acta Derm Venereol 1998;78:76–7. 9498041 10.1080/00015559850135977

[15] Appelgren P Farnebo F Dotevall L. Late-onset posttraumatic skin and soft-tissue infections caused by rapid-growing mycobacteria in tsunami survivors. Clin Infect Dis 2008;47:e11–6. 18549312 10.1086/589300

[16] Pagnoux C Nassif X Boitard C. Infection of continuous subcutaneous insulin infusion site with *Mycobacterium peregrinum*. Diabetes Care 1998;21:191–2. 10.2337/diacare.21.1.191b 9538993

[17] Rolan N Limongi L Putruele AM. *Mycobacterium peregrinum*: an atypical and uncommon mycobacterium. A case report. Arch Bronconeumol (Engl Ed) 2020;56:331–2. 31892448 10.1016/j.arbres.2019.11.008

[18] Sawahata M Hagiwara E Ogura T. Pulmonary mycobacteriosis caused by *Mycobacterium peregrinum* in a young, healthy man. Nihon Kokyuki Gakkai Zasshi 2010;48:866–70. 21141068

[19] Fujiwara K. A case of pulmonary infection caused by *Mycobacterium peregrinum*. IRYO 2008;62:281–4.

[20] Todorova TT Kaludova V Tsankova G. A pulmonary infection caused by *Mycobacterium peregrinum* – a case report. J IMAB 2015;21:1000–2.

[21] Marie I Heliot P Roussel F. Fatal *Mycobacterium peregrinum* pneumonia in refractory polymyositis treated with infliximab. Rheumatology (Oxford) 2005;44:1201–2. 15941728 10.1093/rheumatology/keh700

[22] Jeong YJ Lee KS Koh WJ. Nontuberculous mycobacterial pulmonary infection in immunocompetent patients: comparison of thin-section CT and histopathologic findings. Radiology 2004;231:880–6. 15118112 10.1148/radiol.2313030833

[23] Hagiwara E Sekine A Sato T. Clinical features of pulmonary disease caused by *Mycobacterium fortuitum*. Nihon Kokyuki Gakkai Zasshi 2008;46:788–92. 19044027

[24] Winthrop KL Abrams M Yakrus M. An outbreak of mycobacterial furunculosis associated with footbaths at a nail salon. N Engl J Med 2002;346:1366–71. 11986410 10.1056/NEJMoa012643

[25] Griffith DE Aksamit T Brown-Elliott BA. An official ATS/IDSA statement: diagnosis, treatment, and prevention of nontuberculous mycobacterial diseases. Am J Respir Crit Care Med 2007;175:367–416. 17277290 10.1164/rccm.200604-571ST

[26] Brown-Elliott BA Nash KA Wallace RJ Jr. Antimicrobial susceptibility testing, drug resistance mechanisms, and therapy of infections with nontuberculous mycobacteria. Clin Microbiol Rev 2012;25:545–82. 22763637 10.1128/CMR.05030-11 PMC3416486

